# Conservative Hypomethylation of Mesenchymal Stem Cells and Their Secretome Restored the Follicular Development in Cisplatin-Induced Premature Ovarian Failure Mice

**DOI:** 10.1007/s43032-023-01389-4

**Published:** 2023-11-13

**Authors:** Amira Nabil Salama, Eman Abd El-Fatah Badr, Nanis Shawky Holah, Ahmed A. El Barbary, Mohamed Hessien

**Affiliations:** 1 Directorate of Health Affairs, Joint Regional Laboratories, Shebin El-Koum, Menoufia, 32511 Egypt; 2 Department of Medical Biochemistry, Faculty of Medicine, Menoufia University, Shebin El-Koum City, 32511 Egypt; 3 Department of Pathology, Faculty of Medicine, Menoufia University, Shebin El-Koum City, 32511 Egypt; 4 Department of Chemistry, Faculty of Science, Tanta University, Tanta, 31527 Egypt; 5 Molecular Cell Biology Unit, Division of Biochemistry, Department of Chemistry, Faculty of Science, Tanta University, Tanta, 31527 Egypt

**Keywords:** Premature ovarian failure, Bone marrow-derived mesenchymal stem cells, DNA hypomethylation, Anti-Mullerian hormone, Estradiol

## Abstract

Premature ovarian failure (POF) is one of the main causes of infertility in women under the age of 40 years. Recently**,** epigenetic reprogramming, particularly DNA hypomethylation, has emerged as a promising strategy to enhance the therapeutic potential of mesenchymal stem cells (MSCs). Thus**,** it is crucial to elucidate how far global hypomethylation of MSCs genome can maintain their pluripotency and viability and improve their therapeutic effect in chemotherapy-induced POF mice. Herein, the genomic DNA of bone marrow-derived MSCs (BM-MSCs) was hypomethylated by the DNA methyltransferase inhibitor (5-Aza-dC), and the degree of global hypomethylation was assessed by methylation-sensitive *HepII/MspI* restriction analysis. Next, mildly hypomethylated cells and their secretome were independently transplanted (or infused) in POF mice, established via cisplatin-mediated gonadotoxicity. We found that conservative global hypomethylation of BM-MSCs genome with low doses of 5-Aza-dC (≤0.5 μM) has maintained cell viability and MSCs-specific clusters of differentiation (CD). Engraftment of mildly hypomethylated cells in POF mice, or infusion of their secretome, improved the concentrations of estradiol (E2), follicle-stimulating hormone (FSH), and anti-Mullerian hormone (AMH). Furthermore, mice restored their normal body weight, ovarian size, and ovarian follicle count. This was associated with improved follicular development, where the populations of healthy primordial, primary, secondary, and tertiary follicles were significantly ameliorated, relative to mice transplanted with normally methylated cells. This observational study suggests that transplantation of mildly hypomethylated BM-MSCs cells and their secretome can restore the structural and functional integrity of the damaged ovaries in POF mice. Also, it presents conservative hypomethylation of BM-MSCs and their secretome as a promising alternative to MSCs transplantation.

**Graphical Abstract:**

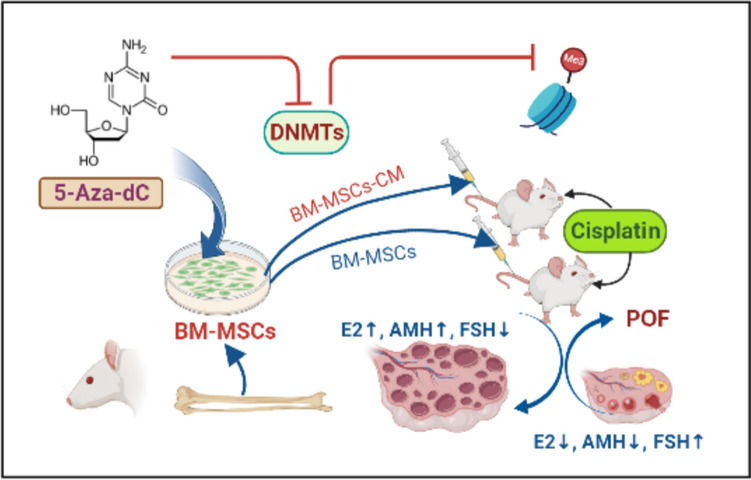

Transplantation of partially hypomethylated BM-MSCs improved the follicular count and integrity in the POF mouse model. Gonadotoxic drug (cisplatin) was used to establish the POF mouse model. In parallel, BM-MSCs were isolated, authenticated, and then incubated with the DNMTs inhibitor (5-Aza-dC). Partially hypomethylated cells and their secretome were independently transplanted into the POF mice, and both the follicular count, ovarian histology, and the serum levels of the fertility-related hormones (E2, AMH, and FSH) were assessed 1 week after transplantation or infusion. Hypomethylated BM-MSCs and their secretome increased the follicular count, increased the number of healthy follicles, and restricted apoptosis of the granulose cells. Also, the hormonal profile was improved compared to their corresponding level in mice transplanted with normally methylated cells.

**Supplementary Information:**

The online version contains supplementary material available at 10.1007/s43032-023-01389-4.

## Introduction

Premature ovarian failure (POF) is one of the most common causes of female infertility. Globally, it affects 3.5% of women, where 11.2% of them with iatrogenic etiology [1]. Clinically, the disease usually manifests amenorrhea, hypergonadotropism, hypoestrogenism, and low levels of the anti-Mullerian hormone (AMH) before the age of 40 years. Although hormone replacement therapy is predominantly used as the first-line therapy, it is unable to solve the problem due to the complexity and irreversibility of POF pathogenesis [2]. This imposed the dispirit need for new therapeutic strategies including a cell-based regenerative approach. Many preclinical studies and clinical trials strongly suggested the promising therapeutic efficacy of stem cell transplantation in the treatment of POF [3–6]. In parallel, other studies suggested the utilization of MSCs-derived exosomes as an alternative and favorable option in regenerative medicine [7]. However, cell transplantation is challenged by their limited in vivo survival and proliferation [8], due to the loss of the extracellular matrix, low nutrients, hypoxia, and activation of host immune response [9, 10]. Accordingly, it is believed that the therapeutic potential of MSCs is largely attributed to their paracrine effect. This raises the notion of preconditioning of MSCs to enhance their in vivo healing performance [11]. This approach has adopted several protocols including exposure of MSCs to sub-lethal stress conditions, like H2O2 [12], hypoxia, heat shock [13], and other means. Additionally, drug-mediated MSCs modifications involving molecular, signaling, or genetic targets were presented as well [14–17]. Normally, natural epigenetic modifications, like DNA methylation/demethylation and histone acetylation/deacetylation, are involved in the constitutional paradigms of stem cell pluripotency and differentiation [18]. Also, epigenetic modulations play a crucial role in the maintenance and transition among different pluripotent stats [19]. Previous reports, for example, revealed that DNA methyltransferase (DNMTs) inhibitors, like 5-Azacytidine (5-Aza C), induced global hypomethylation in MSCs genome and facilitated their osteogenic [20], chondrogenic [21], and adipogenic differentiation [22]. Rather than differentiation, other studies revealed that DNMTs inhibitors maintained MSCs pluripotency via their direct effect on the stemness-related genes [23–25]. Furthermore, epigenetic modifications alleviated the senescence and dysfunction of MSCs cells [26]. Thus, the overall scenario depicts the close association between the epigenome reprogramming of MSCs and the enhancement of their therapeutic potential [27]. However, the contradicting outcomes of hypomethylation (differentiation or pluripotency) predispose challenges in using global hypomethylation modifiers to enhance MSCs differentiation or to maintain their pluripotency. Also, the prevalence of genomic hypomethylation is known to relieve the condensation of chromatin and makes many genes accessible to their transcription factors [28]. This may lead to modifications at the transcriptional and translational levels of MSCs. Accordingly, it is hypothesized that global hypomethylation may improve MSCs therapeutic effect via maintaining their self-renewal property, enrichment of the secretome they release, and the long-term heritable effect of epigenetic modulations. As chemotherapy-induced female infertility represents an important health problem, it is essential to explore the therapeutic potential of hypomethylated BM-MSCs and their secretome in the treatment of POF. To establish this goal, the genomic DNA of BM-derived MSCs was hypomethylated using concentrations range of a DNMTs inhibitor. Next, cells have been investigated for the degree of hypomethylation, cell viability, and the stability of their mesenchymal characteristics. Moreover, both viable hypomethylated cells and their secretome were independently utilized in the treatment of cisplatin-induced POF mice, where the restorative effects were monitored through the follicular development and the associated changes in fertility-related hormones. This new approach may lay a foundation of conservative hypomethylation of MSCs and their secretome in regenerative clinical applications.

## Materials and Methods

### Key Chemicals

Cisplatin was purchased from Mylan, Viatris, PA, USA (Cat. no. 198547); 5-Aza-dC was from Sigma-Aldrich (St. Louis, USA, Cat no. 2353-33-5); *HpaII* and *MspI* restriction enzymes were from Takara, Bio Inc., Japan, (Cat no.1053A and 1150A, respectively); and alkaline phosphatase reagents were from Spectrum Diagnostics, Egypt (Ref. 216-001). Cell culture reagents (Dulbecco’s Modified Eagle *Medium* (DMEM) (Cat. no: 12-707F), minimum essential *medium-α* (MEM-α), fetal bovine serum (FBS), trypsin-EDTA (Cat. no. 17.942E), penicillin/streptomycin, and phosphate buffer saline (PBS) were from Lonza, Pharma Biotechor from BioWhittaker, USA. Phycoerthrin (PE)-conjugated mouse monoclonal antibodies of CD90, and CD34 or FITC-conjugated monoclonal antibodies of CD105 and CD45 were from (Biotechne R&D System, MN, USA).

### BM-MSCs Isolation and Passaging

To isolate BM-MSCs, a rat was sacrificed by cervical dislocation, where bone marrow was collected by flushing of femur and tibia bones with MEMα complete media supplemented with 10% FBS and 1% 100 U/ml penicillin and 100 mg/ml streptomycin mix. Cells were seeded in a T75 tissue culture flask and incubated overnight for cell attachment. Non-adherent cells were removed, and old media were replaced with fresh media twice a week until 90% confluence. For cell expansion, cells were collected and serially expanded to the fourth passage [29].

### Identification of the Phenotypical Markers of BM-MSCs

Fourth passage cells were investigated for the clusters of differentiation. Briefly, cells were collected by trypsinization, centrifuged at 500 × g for 5 min, washed with PBS to remove residual growth factors, resuspended at cell density 4 × 10^5^ cells/ml, and then incubated with phycoerythrin (PE)-conjugated mouse monoclonal CD90 or CD34, or FITC-conjugated CD45 and CD105 antibodies. After 40 min incubation at 4 °C, cells were washed with PBS, resuspended in 300 μl PBS, and then the fluorescence signals were determined using a FACScan flow cytometer (Becton Dickinson, San Jose, CA, USA). The data were analyzed with Cell Quest Software (Becton Dickinson).

### Preparation of BM-MSCs Secretome

Well-authenticated and viable BM-MSCs cells were used to prepare the conditioned media (BM-MSCs-CM), where cells were left untreated or incubated with 2.5 μM, or 5 μM 5-Aza-dC in serum-free media for 24 h. After incubation, media were collected, centrifuged to remove any cell derbies, and utilized in the treatment of POF mice via IV injection as shown below [30].

### Induction of Global Hypomethylation in BM-MSCs Genome

To evaluate the appropriate concentration of 5-Aza-dC to induce sufficient global DNA hypomethylation without disrupting cell viability and MSCs self-renewal properties, MSCs were treated with a range of 5-Aza-dC concentrations (1.25–80 μM) for 24 h. After incubation, cells were observed morphologically under a phase contrast microscope. Also, cell viability and the activity of alkaline phosphatase were accessed as shown below.

### Effect of 5-Aza-dC on MSCs Cell Viability

To determine the cytotoxic limits of 5-Aza-dC on cell viability, 4000 cells in 200 μl of cell suspension/well were cultured in a 96-well plate at cell density 2 × 10^4^ cells/ml in DMEM media containing 0.0, 1.25, 2.5, 10, 20, 40, or 80 μM of 5-Aza-dC. After a 24-h incubation period, cell viability was assessed by MTT assay, following standard protocol [31]. Briefly, after overnight incubation at 5% CO_2_ and 37 °C, with drug-containing media, cells were labeled with 20 μl of MTT solution (5 mg/ml in PBS) per well, followed by 5 min shaking, after which they were incubated in the dark for 4 h. The medium was removed, 150 μl of DMSO was added to dissolve the formazan, and then the absorbance was measured at 570 nM.

### Effect of Hypomethylation on MSCs Phenotypical Markers

To monitor the effect of 5-Aza-dC on BM-MSCs, treated cells were investigated for the expression of CD105, CD90, CD45, and CD34 surface markers, as shown above. As the increased expression and the activity of alkaline phosphatase (ALP) are considered key markers of MSCs, ALP activity was determined, where untreated (control) and treated cells were collected and lysed in 100 μl lysis buffer (50 mM Tris–HCl, pH 8.0, 150 mM NaCl, 1% NP-40, and 0.5% sodium deoxycholate). After sonication, cell lysates were centrifuged, and the supernatant was utilized to determine the activity of the alkaline phosphatase, following the manufacturer’s instructions.

### Assessment of the Degree of Hypomethylation by Methylation Insensitive HpaII/MspI Digestion

Genomic DNA was isolated from BM-MSCs cells or cells pretreated with 2.5, 5, or 10 μM of 5-Aza-dC for 24 h, using a Qiagen DNA-extraction kit (QIAamp mini spin column, Cat. no. 51304). Also, RNA contamination was eliminated by incubating DNA preparations with RNAse A for 4 h at 37 °C. The purity of DNA isolates was assessed by measuring DNA at 260 and 280 nm. Next, the methylation-insensitive digestion method [20] was employed to investigate the methylation level of BM-MSCs genomic DNA after their treatment with 5-Aza-dC. The method is based on the isoschizomers *HpaII* and *Msp1* that recognize and cleave the sequence (5′-CCGG-3′) similarly. Methylation of the internal cytosine residue prevents the cleavage of the recognition site. Briefly, DNA (2 μg) was mixed with 4 μl of *MspI* or *HpaII* buffer and 20 U of the corresponding enzyme. The reaction volume was increased to 40 μl with water, and incubated overnight (16 h) at 37 °C, after which 5 μl of the loading dye was added. The digests were loaded onto 0.8% agarose gel containing ethidium bromide, electrophoresed for 3 h at 80–90V, visualized, and photographed under a UV transilluminator.

### Animal Care, Grouping, and POF Modeling

**Fig. 1:**
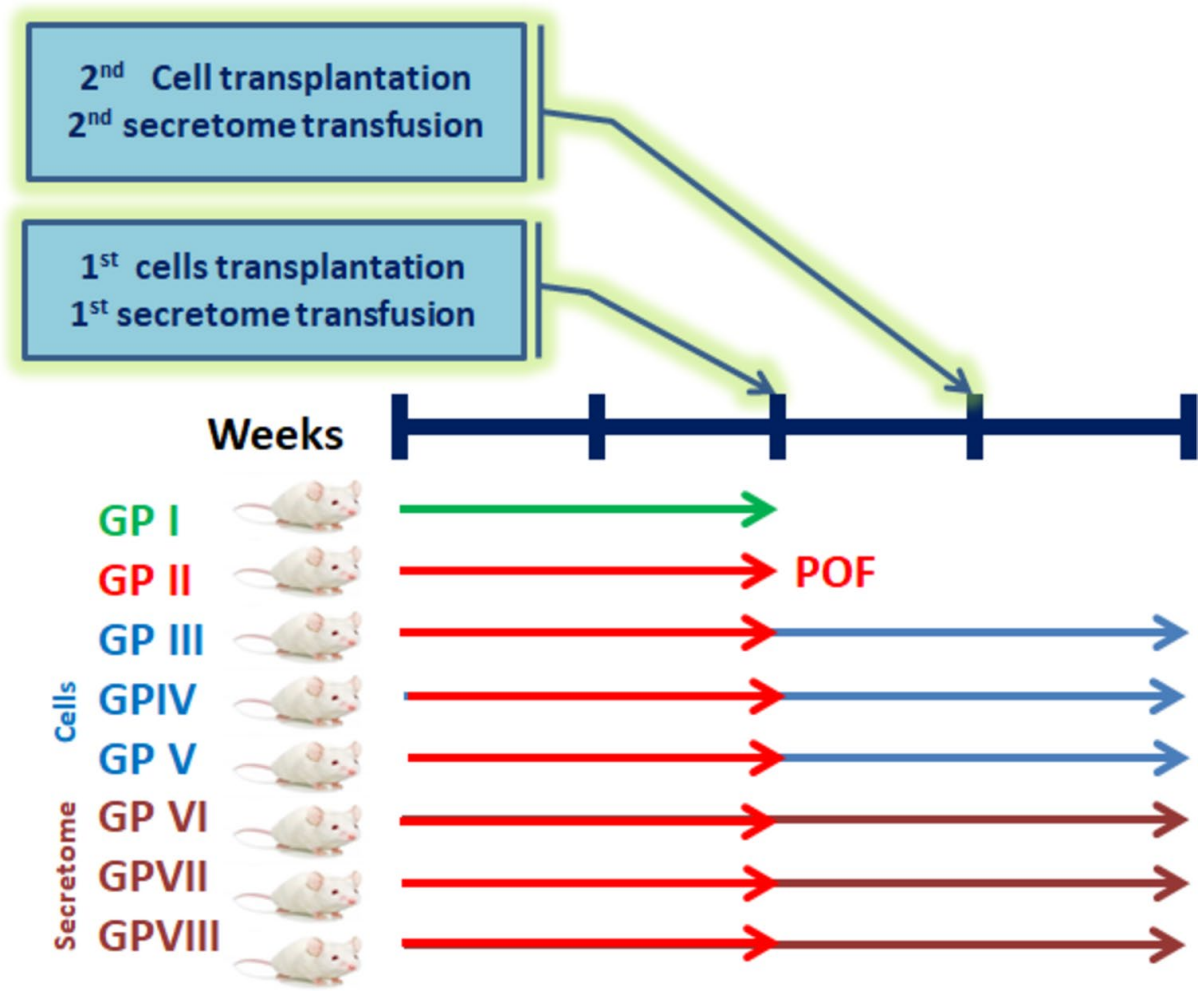
Experimental design of animal grouping and treatment schedule. Mice were assigned to eight groups including normal control group (GpI), POF group (GpII), POF group transplanted with MSCs (GpIII), POF group transplanted with MSCs, which were pretreated with 2.5 μM5-Aza-dC (GpIV) or 5 μM 5-Aza-dC (GpV), POF group treated with conditioned media, which was recovered from normally methylated cells (GpVI) or hypomethylated cells with 2.5 uM (GpVII) or 5 uM5-Aza-dC (GpVIII)

This preclinical study utilized adult female C57BL/6 mice (aged 2–3 months) obtained from the National Cancer Institute, Cairo University, Egypt. Ethical regulations of animal use were strictly applied, and the protocol was approved by the ethical committee, Faculty of Science, Tanta University (IACUC-SCI-TU-0160). Mice were acclimatized for 1 week under a 12/12-h light/dark cycle at an ambient temperature of 21 °C, and they were supplemented with a standard diet and water *ad libitum*. Initially, the development of POF via cisplatin injection [32] was authenticated in small investigative groups (*n*=4 each), in which mice received 2 or 4 mg/kg body weight cisplatin every other day for 15 days (8 ip doses). At the end of treatment periods, mice were sacrificed, and ovaries were recovered for histological examination to assess the ovarian damage and the development of POF signs, as indicated by a decrease in the follicular count, the existence of unhealthy follicles, and the development of apoptosis in granulosa cells. This preliminary step revealed that 4 mg/kg cisplatin generated POF more efficiently than 2 mg/kg cisplatin treatment for a similar period. The main experiment involved 48 healthy adult female mice aged 2–3 months. As shown in the experimental design (Fig. 1), mice were randomly assigned to eight groups (six mice each), including the normal control group (GpI), cisplatin-induced POF group (GpII), POF mice transplanted with BM-MSCs cells (GpIII), and POF mice transplanted with BM-MSCs cells, which were previously incubated with 2.5 μM 5-Aza-dC (GpIV) or 5 μM 5-Aza-dC (GpV). Also, another three POF groups were assigned for treatment with conditioned media. In these groups, mice were injected twice with 400 μl conditioned media, via tail-vein injection 1 week apart. The conditioned media were recovered from normally methylated (GpVI) or hypomethylated cells with 2.5 μM 5-Aza-dC (GpVII) or 5 μM (GpVIII). Mice in all groups had free access to food and water during the study period, where the body weight was monitored just before dosing. In MSCs treated groups, mice were transplanted twice (1 week apart) with 400 μl of BM-MSCs suspended in PBS at a concentration of 1×10^6^ cells/ml, via tail-vein injection. One week after the second transplantation or infusion, mice were euthanized and ovaries were recovered, weighed, and stored at –80 °C until analyzed. Also, blood samples were collected, and the recovered sera were stored at –80 °C until analyzed.

### ELISA for Hormones Assay

To estimate fertility-related hormones, blood samples were left to coagulate, and serum was recovered by centrifugation and kept at −80 °C. Serum levels of E2, FSH, and AMH were estimated by enzyme-linked immunosorbent assay (ELISA), using Cobas Roche Diagnostics GmbH, Mannheim, Germany (Ref. 03000079-190, 11775863 122 and 06331076 190, respectively), following the manufacturer’s guidelines.

### Histological Assessment of Follicle Count and Development

For the ovarian histological analysis, bilateral ovaries were recovered, washed with PBS, and weighed. Portions of tissues were fixed in 10% formaldehyde for 12 h and paraffin-embedded. After sectioning to ~5 μm slices, sections were mounted on a glass slide, stained with hematoxylin and eosin (H&E), following the standard protocol [33]. At least five microscopic fields were examined to determine the follicular count and their histological integrity. In brief, follicles were staged as primordial when an oocyte with a visible germinal vesicle was surrounded by flattened granulosa cells. Also, the developmental stages were defined as primary when the oocyte has a visible germinal vesicle and was surrounded by a single layer of cuboidal germinal cells, secondary when the oocyte with a visible germinal vesicle was surrounded by multiple layers of cuboidal cells, or tertiary when further size enlargement and bigger oocyte. Furthermore, follicles were defined as unhealthy due to the presence of unhealthy oocytes (eosinophilic and shrunken cytoplasm and/or condensed nuclear chromatin), unhealthy granulosa cells with an irregular shape and/or condensed chromatin, the presence of apoptosis, or a combination of unhealthy oocytes and granulosa cells [34].

### Statistical Analysis

Data analysis was performed using the SPSS.26.0 software package (IBM, Chicago, IL, USA). All data are presented as means of at least 3–5 (± standard deviation). The Kolmogorov–Smirnov test was used to assess the normality of the data. Differences between measurements were performed by ANOVA and post hoc Tukey’s honestly significant difference test. Differences were considered significant at *p*<0.05. Graphing was performed using Microsoft Excel, and graphical illustration was performed by BioRender (www.BioRender.com).

## Results

### Lower Concentrations of 5-Aza-dC Maintained MSCs Cell Viability and Their CD Pattern

**Fig. 2:**
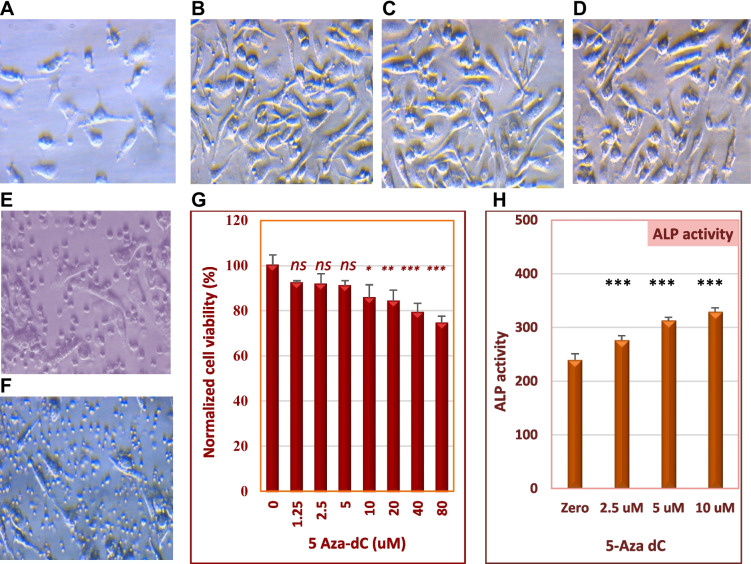
Effect of 5-Aza-dC on BM-MSCs cell viability, phenotypical characteristics, and alkaline phosphatase activity. Cell metabolic activity was determined by MTT assay, after they were treated with a concentration range of 5-Aza-dC, for 24 h. **A** through **E** are representative micrographs of cells after 5 (**A**) and 12 (**B**) days of seeding, cells treated with 2.5 μM (**C**), 5 μM (**D**), 10 μM (**E**), or 20 μM (**F**) of 5-Aza-dC, respectively. Apoptotic changes were observed in cells treated with 10 μM 5-Aza-dC (or higher concentrations), whereas cells treated with lower concentrations (1.25, 2.5, and 5 μM) did not show a significant decrease in their viability, relative to DMSO-treated cells (**G**). Also, treated cells maintained the high activity of ALP (**H**). Bars represent the average of 5 measurements (± SD); *ns*, non-significant; (*) *P*<0.05, (**) *P*<0.01, and (***) *P*<0.001. Comparison between means was performed by one-way ANOVA test

**Fig. 3:**
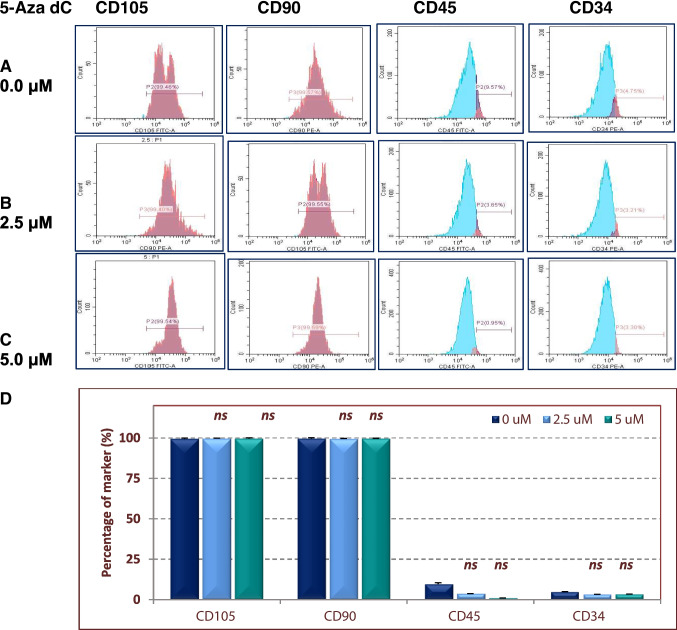
Phenotype characterization of partially hypomethylated MSCs. The expression of CD105, CD90, CD45, and CD34 was analyzed by flow cytometry for normally methylated cells (**A**), cells treated with 2.5 μM (**B**), or 5 μM (**C**) of 5-Aza-dC. No significant differences were observed in the expression of CD markers between DMSO-treated cells and cells treated with 5-Aza-dC (**D**). Bars represent the average (±SD) of three independent measurements, *ns*, not significant. Comparison between means was performed by one-way ANOVA test

Initially, we isolated BM-MSCs cells from a rat’s bone and maintained them until the 4th passage. Cells demonstrated a typical fibroblast-like elongated spindle shape after 3 days of isolation (Fig. 2). Cells of the fourth passage were treated with various concentrations of 5-Aza-dC as shown above. Lower concentrations of 5-Aza-dC (≤5 μM) did not exert any morphological changes, whereas higher concentrations (≥10 μM) led to apoptotic morphological features including cell rounding and detachment. The activity of the mitochondrial dehydrogenase was determined to assess cell viability by MTT assay. The results revealed an insignificant decrease in cell viability when they were treated with 1.25, 2.5, or 5 μM5-Aza-dC (*P*>0.05) relative to the untreated control cells (Fig. 2B–D). However, cell viability progressively reduced (less than 90%) with higher concentrations of the drug (Fig. 2G). Also, we investigated the activity of ALP as a marker of stem cells, where the baseline activity of the ALP observed in DMSO-treated cells has increased by 5-Aza-dC (Fig. 2H). To evaluate the effect of 5-Aza-dC on the expression of MSCs-specific CD markers, cells were treated with drug concentrations that maintained cell viability for 24 h, and then their CD markers were accessed by flow cytometry. As Fig. 3 shows, BM-MSCs were positive for CD105 and CD90 and negative for the hematopoietic lineage markers (CD34 and CD45). Similarly, cells pretreated with 1.25 μM or 5 μM 5-Aza-dC were highly expressing CD105 and CD90 (>99%) and minimally expressing CD45 and CD34, as similar as the untreated cells.

### 5-Aza-dC Induced Global Hypomethylation of MSCs Genome

**Fig. 4:**
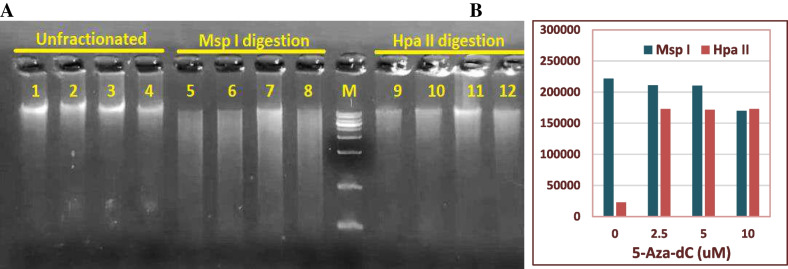
Effect of 5-Aza-dC on the methylation pattern of cell’s genomic DNA. Cells were left untreated or grown for 24 h in the presence of a concentration range of 5-Aza-dC (2.5, 5, or 10 μM). Genomic DNA was isolated and left undigested (lanes1–4), digested with the methylation insensitive cutter *MspI* (lanes 5–8), or with the hypomethylation-sensitive cutter *HpaII* (lanes 10–13) (**A**). **B** represents the intensity of DNA fractionation analyzed by Image J. *MspI* digest generated more DNA fractionation. DNAs derived from 5-Aza-dC treated cells (lanes 10–12) showed a relative increase in *HpaII*-mediated fractionation compared to normally methylated cells (lane 9). M, molecular weight marker (Lambda DNA-Hind III Digest)

To determine the global hypomethylation effect of 5-Aza-dC, genomic DNA preparations from both normally methylated and hypomethylated cells were isolated and separately digested with the isoschizomers, *Msp I* and *HpaII.* The digestion products were resolved by agarose gel electrophoresis to evaluate the cell methylation pattern. As Fig. 4 depicts, when DNAs were digested with the methylation-insensitive CpG restriction enzyme *MspI*, they showed more fractionation than digestion with the hypomethylation-sensitive CpG cutter (*HpaII)*. Furthermore, the degree of DNA fragmentation was higher in cells treated with 5-Aza-dC (lanes 10–12) compared to the DNA derived from untreated cells (lane 9).

### Gonadotoxicity with 4 mg/kg Cisplatin Induced More Efficient POF than 2 mg/kg

As mentioned above, the POF model was developed by cisplatin gonadotoxicity, followed by histological authentication. Ovaries recovered from healthy mice had normal volume and higher counts of primordial, primary, secondary, and tertiary follicles. Also, granulosa cells had no signs of apoptosis. Injection of mice with 2 mg/kg cisplatin (8 doses, day after day) reduced the total number of follicles, but increased the population of unhealthy ones, as indicated by the irregularity of granulosa cells, condensed chromatin, and the development of apoptosis in some fields and existence of normal granulosa in other fields. Moreover, the nuclei of cells in late apoptosis were fragmented resulting in vacuolation and apoptotic bodies’ appearance. Also, ovarian sections showed unhealthy oocytes in most follicles and healthy oocytes in some fields. Notably, the unhealthy oocytes were eosinophilic, with shrunken and condensed nuclear chromatin (Supplementary data, Fig S1). On the other hand, mice intoxicated with 4 mg/kg cisplatin, for a similar period, demonstrated severe reduction in the ovarian volume associated with atrophy. Also, we observed a massive reduction in the follicular count, severe apoptosis of the granulosa cells, and fragmentation of the nucleus of follicles resulting in vacuolation and apoptotic bodies’ appearance. Furthermore, the cytoplasm of the oocytes was severely eosinophilic and shrunken, and the nuclei showed condensed chromatin. These observations suggested that 4 mg/kg body weight efficiently generated the POF model, especially no animal mortality was associated with this dose.

### Cisplatin Reduced Both Body Weight and Ovarian Weight in POF Mice

**Fig. 5:**
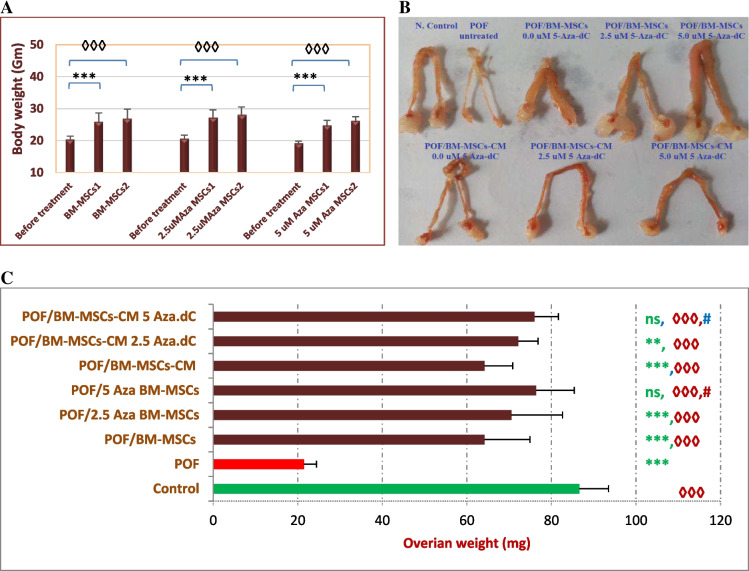
Effect of the engraftment of hypomethylated MSCs and their secretome on POF mice body and ovarian weight. **A** shows the changes in the body weight after the first and the second transplantation. **B** is a representative photo of ovaries recovered from healthy, POF, or treated groups. **C** depicts the average ovary weight. Bars represent the average (±SD). (*, ◊, or #) refer to significant differences between the indicated group versus color matched reference group. Ns, not significant. Comparison between means was performed by one-way ANOVA test

During the intoxication and treatments, we monitored the changes in animal body weight and ovarian weight as well. Healthy mice (Gp1) demonstrated slight weight gain, where their initial weight (29.1±2.75 gm) increased to 30.74±2.09 gm (*P*>0.05) at the end of the intoxication period (Supplementary data, Fig. S2). The body weight of POF mice (generated by 2 mg/kg or 4 mg/kg cisplatin) significantly reduced (*P*<0.01, *P*<0.01). In parallel, the ovary weight of 4 mg/kg cisplatin-induced POF mice (Gp2) dramatically decreased to 21.49 ±2.94 mg versus 86.64±6.87 mg in healthy mice (Fig. 5).

### Hypomethylated MSCs and Their Secretome Similarly Improved Animal Body Weight and Ovarian Weight

Mice transplanted with normally methylated MSCs (Gp III), cells pre-hypomethylated with 2.5 μM (Gp IV), or 5 μM 5-Aza-dC (GP V) demonstrated significant improvement in their average body weight, estimated 1 week and 2 weeks after the first transplantation, compared to the POF group (GPII) (*P*<0.001, *P*<0.001, respectively). Additionally, the ovarian weight was significantly increased when mice were treated with hypomethylated cells or their secretome, compared to the corresponding normally methylated cells or their secretome (Fig. 5).

### Hypomethylated Cells and Their Secretome Improved the Number and Quality of the Ovarian Follicles

**Fig. 6:**
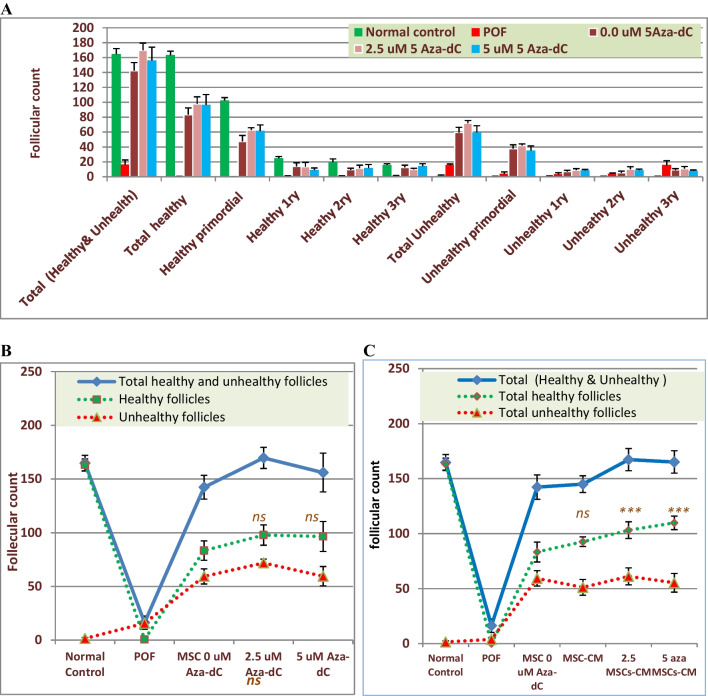
Effect of the engraftment of hypomethylated MSCs transplantation and their secretome on the differential follicular count in POF mice. **A** represents the count of the total number of follicles, primordial, primary, secondary, and tertiary follicles, determined in at least in 4–6 H&E-stained sections recovered from healthy, POF, or treated mice. **B** and **C** depict the changes in the numbers of healthy and unhealthy follicles in animals treated with cells (**B**) or their secretome (**C**). Bars or data points represent the averages of follicle number (±SD). (***) refers to a significant difference (*P*<0.001) between the indicated group versus the corresponding group treated with unmodified cells or their secretome. *ns* refers to insignificant difference. Comparison between means was performed by one-way ANOVA test

**Fig. 7:**
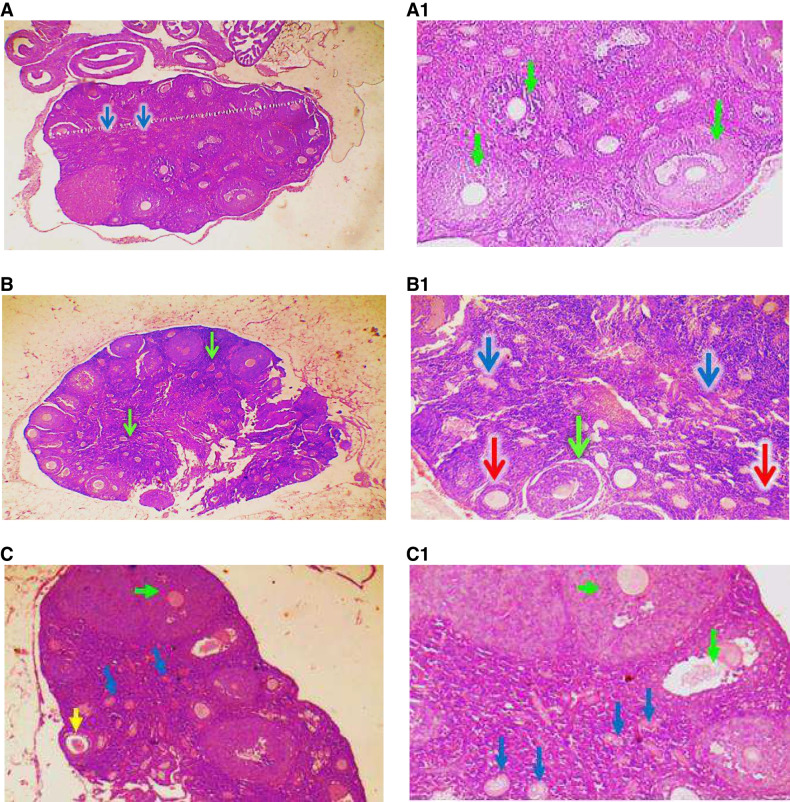

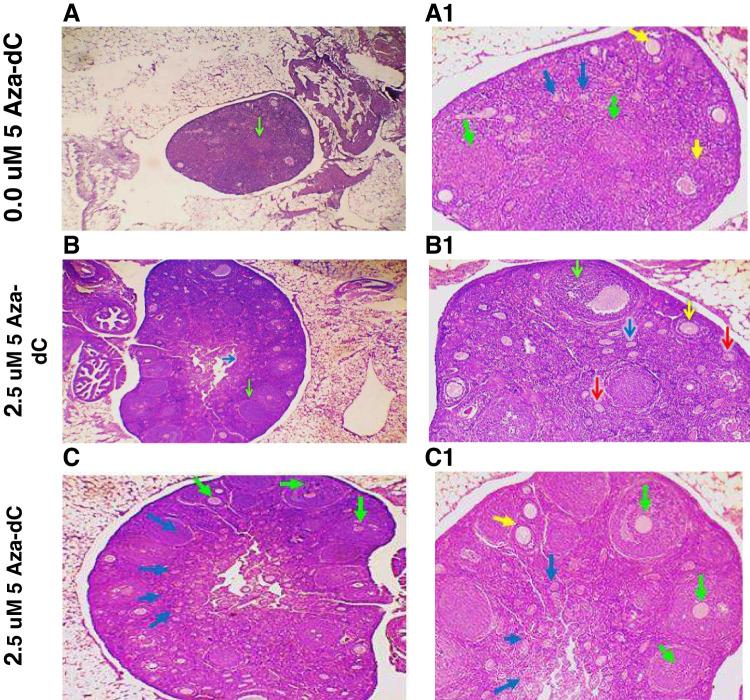
Ovarian histological integrity was restored in POF mice after treatment with hypomethylated cells and secretome. Transplantation of hypomethylated MSCs (7**I**) and transfusion of their secretome (7**II**) improved the ovarian architecture in POF mice. Representative mouse ovarian tissue image stained with H&E section derived from mice transplanted with normal cells (A and A1), cells hypomethylated with 2.5 μM 5-Aza-dC (B, B1), or cells hypomethylated with 5 μM 5-Aza-dC (C, C1). 7**II** includes representative stained sections recovered from POF groups transfused with the secretome of normally methylated cells (**A, A1**), cells hypomethylated with 2.5 μM (**B, B1**) or 5 μM (**C, C1**) 5-Aza-dC. Yellow, red, green, and black arrows indicated primary, secondary, tertiary, and apoptosis in stroma cells, respectively.

Differential count of the growing follicles demonstrated that the total follicle count per H&E sections derived from healthy mice was 164.7± 6.1 and dramatically decreased to 16.33±2.2 in POF mice (Gp2), where most follicles (15.67±1.47) were underdeveloped (Fig. 6A). The follicle counts in mice transplanted with normal cells, cells hypomethylated with 2.5 μM, or 5 μM 5-Aza-dC were 142.3±11.1, 169.7±9.8, and 156±18.0, respectively. These treatments were associated with a significant increase in the population of healthy follicles (83.3±9.0, 97.8±9.5, and 96.5±14.0, respectively) (Fig. 6B). To further explore the healing potential of hypomethylated cells-derived secretome, another set of POF groups were treated with secretome derived from normal cells, cells pretreated with 2.5 μM 5-Aza-dC, or 5 μM 5-Aza-dC. A similar improvement pattern was observed 1 week after the second infusion. The total counts of healthy and unhealthy follicles increased to 145±7.6, 92.7±4.4, and 51.2±7.3, respectively, in mice infused with secretome derived from normal cells. More significant amelioration was seen when mice were treated with modified cells derived secretome (Fig. 6C), where hypomethylated cells-derived secretome was associated with higher numbers of healthy follicles (103.2±6.6, 109.8±6.2), compared to 92.7±4.4 in mice injected with normal cell secretome. Histologically, transplantation of POF mice with normal BM-MSCs led to the development of healthy primordial, primary, and secondary follicles, but unhealthy tertiary follicles with shrunken cytoplasm, condensed chromatins, and multiple apoptosis. Also, some sections showed abnormal secondary follicles with mild apoptosis and oocyte with condensed chromatins (Fig. 7I). Hypomethylated cells improved the ovarian size and increased follicle count. This was associated with the development of normal primordial and primary follicles. However, the tertiary follicles were underdeveloped (Fig. 7 D and E). Injection of MSCs secretome in POF mice showed an increased size of ovary and number of follicles with an increased number of healthy follicles, especially the tertiary (green arrow) and primordial (blue arrow). Also, no apoptosis was observed in the granulosa cells (Fig. 7II).

### Hypomethylated BM-MSCs Improved Fertility-Related Hormones

**Fig. 8:**
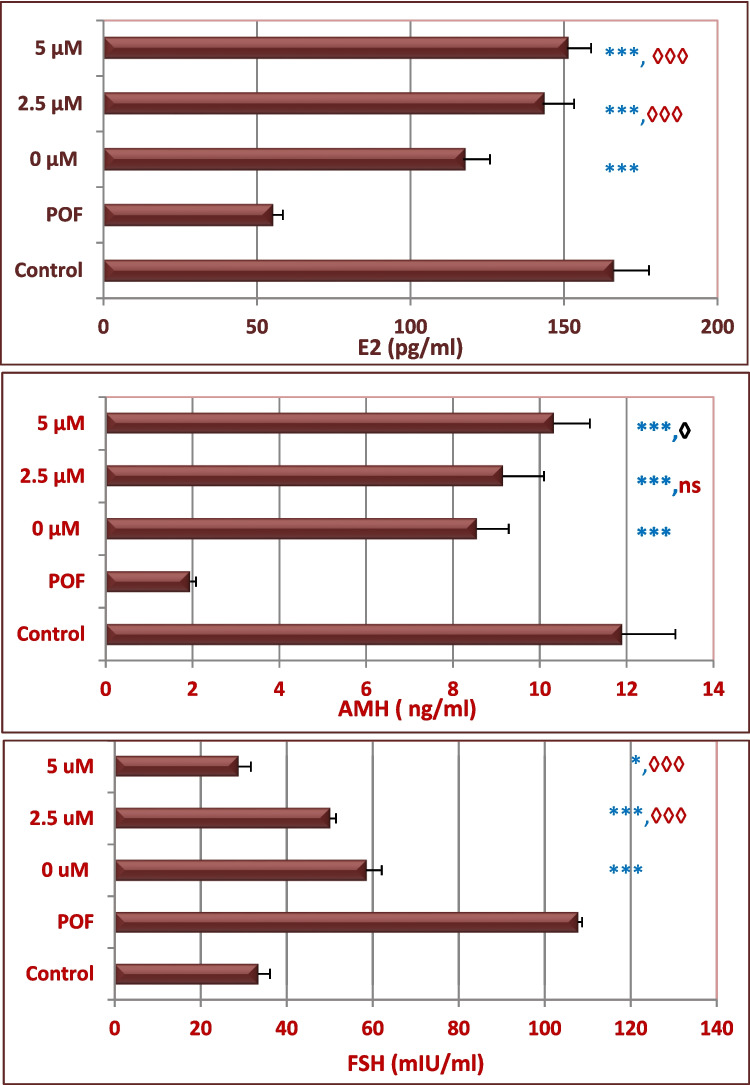
Improvement of the endocrine functions after the engraftment of hypomethylated MSCs. Normally methylated cells and cells hypomethylated by 2.5 or 5 μM 5-Aza-dCwere transplanted twice (1 week apart) in POF mice. One week after the second transplantation, the serum levels of E2, AMH, and FSH hormones were estimated. Significant ameliorations in hormone level were observed in mice transplanted with normal cells, and more improvements were observed in mice transplanted with 2.5 μM or 5 μM 5-Aza-dC modified cells relative to POF mice or mice treated with normally methylated cells. Abbreviation: E2 estradiol, AMH anti-Mullerian hormone, FSH follicle stimulating hormone. (* or ◊) refer to a significant difference between the indicated group versus POF (GpII) or mice treated with normally methylated BM-MSCs (Gp III), respectively. Statistical comparison between means was assessed by ANOVA test.

Next, we estimated the serum levels of fertility-related hormones. The baseline levels of E2, FSH, and AMH assayed in healthy mice (GpI) were deteriorated in POF mice (GpII) as indicated by the reduction of E2 and AMH associated with the over secretion of FSH. One week after cell transplantation, the serum levels of FSH and E2 were significantly decreased and AMH was significantly increased (*P*<0.001) when mice were transplanted with MSCs relative to the POF mice (GpII) (*P*<0.001). Also, more amelioration was observed in the hormonal profile in mice transplanted with hypomethylated cells relative to the normally methylated mice (Fig. 8).

## Discussion

This observational study revealed that conservative global hypomethylation of BM-MSCs genome has maintained their cell viability and phenotypical characteristics. Also, globally hypomethylated MB-MSCs and their secretome effectively restored the normal body weight and ovary weight of POF mice. More interestingly, they relieved the ovarian damage and improved follicular development, where they increased the population of healthy follicles in treated mice. Physiologically, the levels of fertility-related hormones, E2, FSH, and AMH, were restored. Initially, we induced global hypomethylation in MSCs genome by using 5-Aza-dC, rather than 5-Aza-C, as the former is 10-fold less cytotoxic [35]. In agreement with previous studies [27], 5-Aza-dC induced a progressive cytotoxic effect. However, BM-MSCs cell viability was minimally reduced when they were treated with concentrations ≤5 μM. The cytotoxic effect of higher doses led to the development of apoptotic morphological features in BM-MSCs. In parallel, the activity of alkaline phosphatase, as a pluripotency marker [36], was elevated as well. More importantly, the expression pattern of MSCs-specific CD markers (CD105 and CD90) as well as the hematopoietic markers (CD45 and CD34) of lightly-hypomethylated cells was quite similar to normal cells, confirming the preservation of their mesenchymal characteristic [37]. Long-term exposure of MSCs or their incubation with higher concentrations (>10 μM) of 5-Aza-dC, reported in similar studies, triggered the mesenchymal-epithelial transition (EMT) and subsequent differentiation commitments towards chondrogenic, osteogenic, or adipogenic [38–40]. This scenario may predict the detrimental effect of global hypomethylation on MSCs fate. Also, it may suggest the dose-dependent biphasic role of global hypomethylation, whether it acts as a tool for MSCs preconditioning, with lower concentrations, or differentiation enhancer, with higher concentrations of DNMT inhibitors. Partial global hypomethylation, the new approach we present, is considered a regulatory mechanism of pluripotency-related genes [41, 42] and a preliminary reprogramming method to enhance BM-MSCs paracrine-mediated healing effect.

Cisplatin is a well-reported gonadotoxic chemotherapy, where it damages the granulosa cells, which represent the millstone of normal follicular functions and development. Although the mechanism of cisplatin-induced ovarian damage is not fully understood, the production of reactive oxygen species (ROS) and depletion of the antioxidants may predispose its gonadotoxic effect [43]. This suggestion was supported by the ameliorative effect of antioxidants, like resveratrol, which significantly improved the ovarian reserve and increased the AMH level in the POF rats [44]. Alternatively, other studies suggested that cisplatin gonadotoxicity is attributed to the overactivation of the dormant primordial follicles through PTEN/AKT/FOXO3a [32]. The present work has investigated two doses of cisplatin, 2 and 4 mg/kg body weight. Based upon the depletion of the follicular reservoir and the follicular developmental stages, mice intoxicated with 2 mg/kg were associated with underdeveloped follicles and oocytes and the presence of healthy oocytes in some fields. Moreover, the unhealthy oocytes were eosinophilic, with shrunken cytoplasm and/or condensed nuclear chromatin. The higher dose of cisplatin (4 mg/kg body weight), in contrast, effectively generated typical physiological and histological features of POF as indicated by weight loss, reduction of ovary weight, and low levels of E2 and AMH due to the development of massive apoptosis of the granulosa cells, in which both hormones are synthesized. Additionally, the feedback mechanism of E2 reduction stimulates the pituitary gland to increase the gonadotrophic hormones [45]. The underdevelopment of all stages of follicle maturation was the hallmark of the model we utilized. These POF-related pathological features were repeatedly challenged with stem cell therapy, where the literature has accumulated many preclinical and clinical studies concerned with the utilization of different types of MSCs in the treatment of POF in both animal models and humans; however, the underlying mechanisms are not fully understood. In this regard, several scenarios were suggested including stem cell-mediated antioxidant effect, drug-mediated upregulation of survival-related genes (like SURVIVI and BCL2), downregulation of the apoptosis-related genes (like CASPASE-3 and CASPASE-9), and/or the regulatory role of some fertility-related receptors [3]. Furthermore, other reports involved the role of anti-inflammatory proteins released by MSCs and the associated modulation of the PI3K/Akt pathway [46]. Herein, the therapeutic potential of MSCs was taken one step further, where BM-MSCs cells were partially hypomethylated and both reprogrammed cells and their secretome were utilized in the treatment of POF mice. One week after the second cell transplantation, or secretome infusion, the pathological conditions were remarkably relieved, as indicated by the restoration of ovarian weight, the total follicular count, and the improvement in the quality of follicles and ova in different stages. In parallel, POF mice regained the normal concentrations of E2, AMH, and FSH. The associated existence of apoptosis-free granulosa cells and the reduction of FSH were good indicators of normal folliculogenesis. Notably, hypomethylated cells and their secretome demonstrated more improvement, compared to the normally methylated cells. Also, the secretome of the hypomethylated cells demonstrated a significant improvement in the number of well-developed follicles compared to the corresponding treatment with the normally methylated cells. Similar studies showed that the transplanted MSCs are homed on the ovarian stroma, rather than on the follicles, and the observed regenerative effect may occur via the paracrine effect of the transplanted cells through the improved vascular network in the ovary, particularly in the theca cell layer [47]. In the same context, several genes associated with ovarian function are regulated by epigenetics, like AMH [48], neuronatin (NNAT) [49], DNA methyltransferases (Dnmt1, Dnmt3a, Dnmt3b, Dnmt3L) [50], or DENN domain containing 1A (DENNDIA) [51]. These genes are known to be highly methylated in POF. It is expected that the transplanted cells and their secretome may include residual amounts of the hypomethylating agent that reversed the abnormal expression of these genes. Also, hypomethylated cells and their secretome may contain higher levels of stimulatory factors that maintain the granulosa cell proliferation and functions including the expression of the steroidogenic enzymes [7].

Although MSCs transplantation is considered as a valuable strategy for treating POF, the consequences of global hypomethylation of their genome are not fully understood. It is not known how partial genomic hypomethylation qualitatively or quantitatively affected the secretome they release. Future investigations may target the impact of hypomethylation on the transcriptome and proteome of MSCs and define their targets in regenerating the damaged ovarian to achieve pregnancy. Moreover, more investigations are needed to explore the molecular mechanisms that derived the follicular development and antiapoptotic effect on the granulose cells.

In conclusion, chemotherapy-mediated cancer treatment has been well documented to have an adverse effect on female fertility leading to POF. This observational study demonstrated that transplantation of wisely hypomethylated BM-MSCs may offer a new strategy to restore normal folliculogenesis in POF mice. Hypomethylation of MSCs with low concentrations of 5-Aza-dC maintained cell viability and mesenchymal characteristics. Both hypomethylated cells and their secretome restored the normal developmental stages of follicles 1 week after their delivery in POF mice. Also, the associated improvement of the fertility-related hormones predicts the functional integrity of the developed follicle. Moreover, transfusion of hypomethylated BM-MSCs-derived secretome is presented as a preferable cell-free therapeutic strategy due to the soluble growth factors, anti-inflammatory, cytokines, and miRNAs it contains.

### Supplementary Information

ESM 1 (DOCX 698 kb)

## Data Availability

https://zenodo.org/record/8322881

## Abbreviations

CD: Clusters of differentiation
DNMTs: DNA methyltransferases
E2: Estradiol
AMH: Anti-Mullerian hormone
FSH: Follicle stimulating hormone
