# Supplementary material for: Conservative Hypomethylation of Mesenchymal Stem Cells and Their Secretome Restored the Follicular Development in Cisplatin-Induced Premature Ovarian Failure Mice
Source: Reprod Sci. 2023 Nov 13;31(4):1053–68. doi: 10.1007/s43032-023-01389-4 (PMC10959784; doi:10.1007/s43032-023-01389-4)
Supplement: Supplementary file 1 — (DOCX 698 kb) [file 43032_2023_1389_MOESM1_ESM.docx]

**Supplementary figures**

**Fig. S1**

| **A (40X)** | **B (40X)** | **C (40X)** |
| --- | --- | --- |
| 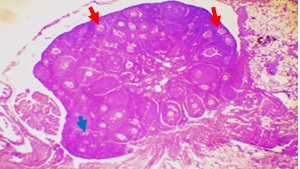 | 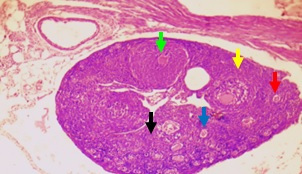 | **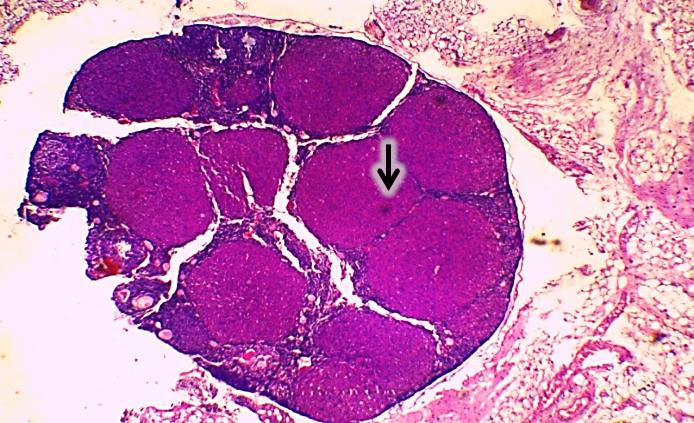** |
| **A1 (200X)** | **B1 (200X)** | **C1 (200X)** |
| **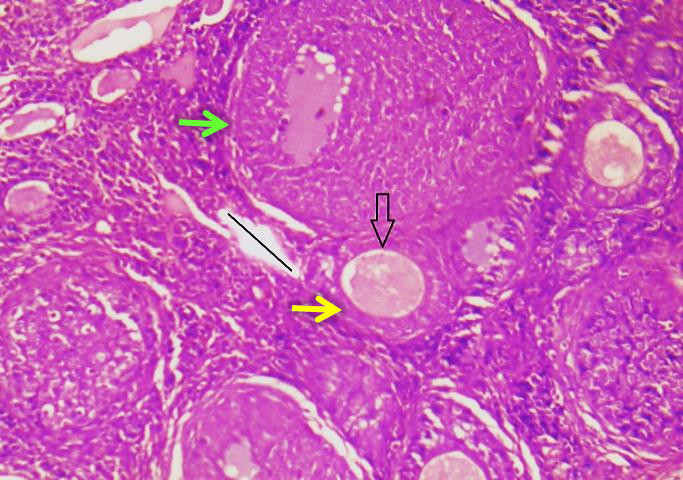** | *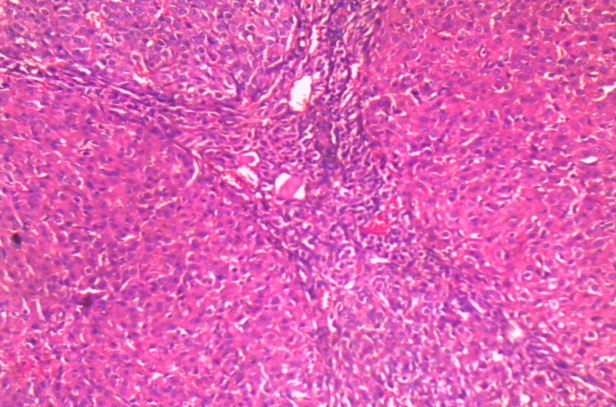* | 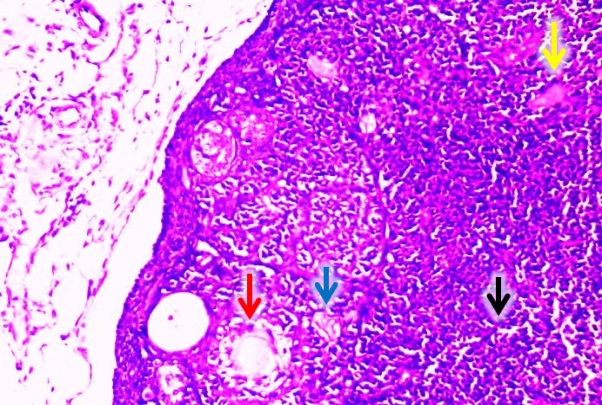 |

**Fig. S1.** Establishment of POF model. H&E staining of ovarian sections derived from healthy group (A, A1), POF mice generated by 2 mg/kg body weight cisplatin (B, B1), and POF mice intoxicated with 4 mg/kg body weight cisplatin (C, C1). Healthy animals demonstrate normal ovarian size and normal follicular count. Also, they showed healthy follicles in control group (A1). POF mice had a massive reduction in the number of follicles, development of few apoptosis in the granulomas cells in when POF was induced by 2 mg/kg cis (B1). Ovarian atrophy, severe reduction in the follicle member, abnormal oocyst and severe apoptosis in POF mice receiving 4 mg/kg (C1).

**Fig. S2**

| **A** | **B** | **C** |
| --- | --- | --- |
| *ns* |  |  |

Fig. S2: A, B, and C show the changes in the body weight of healthy group, animals received either 2, or 4 mg/kg body weight, respectively. The body weight of POF mice was significantly decreased compared to the corresponding initial weight.
